# Neuroplasticity in the Pathology of Neurodegenerative Diseases

**DOI:** 10.1155/2020/4245821

**Published:** 2020-05-27

**Authors:** Jolanta Dorszewska, Wojciech Kozubski, Wioletta Waleszczyk, Matthew Zabel, Kevin Ong

**Affiliations:** ^1^Laboratory of Neurobiology, Department of Neurology, Poznan University of Medical Sciences, Poznan, Poland; ^2^Chair and Department of Neurology, Poznan University of Medical Sciences, Poland; ^3^Laboratory of Visual Neurobiology, Department of Neurophysiology, Nencki Institute of Experimental Biology, PAS, Warsaw, Poland; ^4^College of Medicine, California Northstate University, CA, USA; ^5^General Medicine Department, Armadale Health Service, Mount Nasura, Australia

The phenomenon of neuroplasticity was first described about 50 years ago. Neuroplasticity is a primary property of the nervous system that has been widely observed in both physiological and pathological conditions. Neuroplasticity in physiological conditions includes developmental plasticity, learning and memory, compensatory plasticity, and repair of the adult brain. Neuroplasticity in pathological conditions include plasticity after injury and removal of brain tumor, stroke, epilepsy, and neurodegenerative diseases such as Alzheimer's disease, Parkinson's disease, and Huntington's disease. The current special issue is primarily concerned with the pathogeneses of common neurodegenerative diseases and their effects on neuroplasticity.

It is known that the central changes that occur during a stroke vary widely. Regeneration after ischemic injury and recovery of lost functions can be attributed to the plasticity of neurons which enable their ability to reorganize and rebuild the brain. These neuroplastic changes which include gene expression and possession of specific genetic variants are influenced by environmental factors and epigenetic mechanisms. Neuro-, glio-, and angiogenesis processes, molecular changes occurring after stroke such as synaptic plasticity and axon growth, are discussed in the article by J. Dąbrowski et al.

Many rare diseases are due to disorders at the genetic level such as Waardenburg syndrome (WS). WS is one of the most common forms of deafness with heterogeneity of loci and alleles and variable expression of clinical features. In this issue, W. Li et al. described fourteen mutations in Chinese patients with WS, including c.808C>G, c.117C>A, c.152T>G, c.803G>T, c.793-3T>G, and c.801delT on *PAX3*; c.642_650delAAG on *MITF*; c.122G>T and c.127C>T on *SOX10*; c.230C>G and c.365C>T on *SNAI2*; and c.481A>G, c.1018C>G, and c.1015C>T on *EDNRB*. Importantly, three CNV variants were *de novo* and five *EDNRB* mutations were first associated with WS type 1 heterozygous, with a detection rate of 22.2%. These studies suggest that *EDNRB* mutations should be considered as another common pathogenic gene in WS type 1. More information can be found in the articles by W. Li et al.

At the cellular level, synaptic plasticity plays an important role in maintaining neuronal connections. Synaptic plasticity leads to changes in the structure of synapses and dendritic spines, synaptogenesis, and axonal modification. Disturbed contact between neurons leads to senile changes and elderly diseases such as dementia. Common causes of dementia include Alzheimer's disease, vascular dementia, Parkinson's disease dementia, dementia with Lewy bodies, and frontotemporal dementia. These diseases are characterized by impaired cognitive function as well as personality and emotional changes, including psychopathological disorders. The clinical picture may also include other neurological symptoms such as parkinsonism.

Neuroplasticity at molecular and cellular levels occurs as short-term plasticity (STP), long-term potentiation (LTP), and long-term potentiation depression (LTD). Activation of the NMDA receptor especially in the CA1 region of the hippocampus is necessary for the induction of LTP, even though LTP may be induced in almost all brain structures. GABAergic inhibitory transmission is another important molecular process involved in synaptic plasticity, learning, and memory. Abnormal GABAergic transmission is involved in the pathophysiology of fragile X syndrome (FXS), an inherited neuropsychological disease caused by silencing of the *fmr1* gene and deficiency in the mental retardant protein fragile X (FMRP). Patients with FXS show neuronal changes that lead to severe intellectual disability. More information can be found in the articles by F. Gao et al.

Molecular factors responsible for synaptic transmission disorders include *β*-amyloid deposition; tau aggregation forming neurofibrillary tangles; *α*-synuclein accumulation; growth factors such as BDNF, NGF, and GDNF impaired levels; and disorders of the immune system. Experimental studies using glial cell lines designed to secrete high levels of neurotrophic factor (human ARPE-19 cells) implanted in the rat striatum showed a beneficial effect on GDNF distribution throughout the striatum and their neuroprotection. It seems that GDNF has effective action not only in experimental animals but also in patients with Parkinson's disease, stroke, epilepsy, and Huntington's disease. More information may be found in the article by D. F. Emerich et al.

Another molecular factor involved in the regulation of various processes in neurons and synaptic plasticity is glycogen-3*β* synthase kinase (GSK-3*β*). Altered GSK-3*β* expression or deletion in mice may cause behavioral and cognitive abnormalities. Disturbed GSK-3*β* activity may lead to the development of synaptic plasticity dysfunction and development of neuropsychiatric and neurodegenerative disorders. For more information on GSK-3*β* contribution to neuropsychiatric and neurodegenerative disorders, refer to the article by T. Jaworski et al.

Among the factors affecting synapse plasticity and neurogenesis, astroglia need to be considered. Astrocytes are the most numerous glial cells and play a key role in brain function. Yet little research has been done on their role in neurodegeneration. Research done so far on experimental models indicates the importance of cross signaling pathways between astrocytes and dopaminergic neurons. In Parkinson's disease, astrocytes are involved in neuronal survival and viability. More information on the neuroprotective potential for normalizing astrocyte function in the animal model of Parkinson's disease is available in the article by L. Udovin et al.

Neuroplasticity not only rebuilds brain function but also repairs developmental disorders and is responsible for learning and memory. It appears in response to various internal and external insults and factors, including age-dependent neurodegenerative diseases and pharmacological treatment.

It is known that the repair of brain damage depends on the degree and extent of central lesions. This is an important consideration in the neurorehabilitation and neuropsychological treatment of patients with central nervous system disorders like Alzheimer's disease. Recent studies suggest that intensive and cognitively demanding physical exercise programs are capable of inducing plastic brain changes in Parkinson's disease. The article by E. Palasz et al. elaborates on physical training that provides protection for dopaminergic neurons in rodent parkinsonism models produced by neurotoxins.

We should also mention innovative therapeutic methods to improve neuroplasticity such as deep brain stimulation (DBS). DBS is a recognized therapy for Parkinson's disease. There is evidence that DBS exerts dopaminergic neuroprotection in animal models with disease-modifying effects. Rodent studies have shown an increase in cholinergic neurotransmitters, hippocampal neurogenesis, synaptic plasticity, and reduction of *β*-amyloid plaques with DBS. The effectiveness of DBS is currently being evaluated in patients with mild Alzheimer's disease 65 years of age and older.

Finally, with better understanding of brain plasticity mechanisms in neurodegeneration, more effective therapies and improvement in the quality of life for neurological patients can be achieved.

*Jolanta Dorszewska* *Wojciech Kozubski* *Wioletta Waleszczyk* *Matthew Zabel* *Kevin Ong*

